# Exploring the consequences of nurses’ involvement in disaster response: findings from a qualitative content analysis study

**DOI:** 10.1186/s12873-024-00994-z

**Published:** 2024-04-29

**Authors:** Jamileh Farokhzadian, Parvin Mangolian Shahrbabaki, Hojjat Farahmandnia, Gülcan Taskiran Eskici, Faezeh Soltani Goki

**Affiliations:** 1 Nursing Research Center, Kerman University of Medical Sciences, Kerman, Iran; 2 Health in Disasters and Emergencies Research Center, Institute for Futures Studies in Health, Kerman University of Medical Sciences, Kerman, Iran; 3 Department of Nursing Administration, Faculty of Health Sciences, Ondokuz Mayis University, Samsun, Turkey

**Keywords:** Nurses, Disaster response, Consequence, Qualitative study, Content analysis

## Abstract

**Background:**

The presence of nurses during disasters can lead to many consequences. Understanding the nurses’ experiences of these consequences can provide valuable insights. Therefore, this study was conducted to explore the experiences of Iranian nurses regarding the consequences they faced when being present during disasters.

**Methods:**

This qualitative descriptive study employed a content analysis approach to examine the topic at hand. 20 nurses working in the emergency units of Kerman hospitals were selected through purposive sampling. In-depth semi-structured interviews were conducted to collect the data. The data were analyzed using qualitative content analysis proposed by Graneheim and Lundman. MAXQDA 10 was used to manage data.

**Results:**

After analysis of the interviews, two main categories were identified: overlapping of job frustrations and acquiring experience in difficult conditions. The main category of acquiring experience in difficult conditions comprised the following subcategories: improved quality of care, experience and dedication in fulfilling the role, reduced stress, occupational motivation and enthusiasm, increased self-confidence, and improved social status. On the other hand, the category of overlapping of job frustrations also consisted of the subcategories of physical strength deterioration, psychological and emotional distress, discontinued from supports, feelings of hopelessness, increased exposure to violence and aggression, and occurrence of family problems.

**Conclusion:**

Being present during disasters and obtaining the necessary competencies can have positive consequences that serve as turning points in the personal and professional growth of nurses. Additionally, favorable outcomes can help nurses cope with adverse circumstances. Managers can utilize the findings of this study to develop strategies aimed at reducing negative outcomes and enhancing positive ones among nurses during disasters.

## Introduction

The presence of nurses during disasters has significant impacts that highlight their crucial role in such situations. Their presence ensures immediate medical care for those affected, reducing casualties and potentially saving lives [1]. Nurses assess and prioritize patients, efficiently allocating resources to provide prompt care to those in critical needs. Additionally, they offer emotional support to survivors, providing reassurance and alleviating anxiety in chaotic circumstances [2]. With their expertise in psychological first aid and counseling, nurses help victims cope with the psychological aftermath of the disaster [3]. Nurses also play a vital role in coordinating efforts with other healthcare professionals, emergency response teams, and community organizations, facilitating a holistic approach to disaster management [4]. In a systematic review by Timalsina’s, highlights the presence of nurses during disasters that contributes to reducing the adverse effects of such events. Their provision of immediate medical care, emotional support, coordination efforts, and contribution to disaster preparedness all demonstrate their crucial role in ensuring a comprehensive and effective response to emergencies [5].

Disasters can have a profound impact on healthcare systems and the nurses who work within them [6]. Nurses may encounter a range of challenges at the personal, professional, organizational and family levels during disasters. These challenges can have adverse consequences for their well-being [7]. A Croatian study by Civljak et al., it was found that 70% of nurses reported experiencing physical and psychological injuries during disasters [8]. According to Vagni et al. study from Italy the physical and mental vulnerability of nurses in the work environment is further intensified during disasters, leading to irritability, difficulty sleeping, intrusive thoughts, reduced activity level, emotional numbness, and emotional exhaustion [9]. Therefore, nurses are more exposed to adverse consequences during disasters than other healthcare professionals. Wang et al. highlighted factors that can help reduce the impact of disasters on nurses [10]. These factors can include creating positive relationships with nurses based on respect, understanding and reassuring them [11]. Furthermore, Said et al., in a review study, reported that by increasing the competencies of nurses in disaster response, they could gain positive and desirable achievements when present during disasters [3].

A study by Alharbi in Saudi Arabia reported that identifying the consequences that nurses face during disasters can help to properly prepare and effectively train nurses and generally improve health and minimize injuries during disaster [12]. A meta-synthesis study was conducted by Ma on the experiences of nurses involved in disasters. Researchers stated that health managers need to discover and understand the consequences of disasters for nurses more deeply in order to meet the needs of nurses, support programs and improve the quality of care during disasters [13].

Although several reports have highlighted the negative consequences of the nurse’s presence, they is still a lack of comprehensive understanding of these issues [14, 15]. A review of the conducted studies showed that these studies focused on various aspects of the roles, challenges and preparation of nurses in responding to disasters or only examined the knowledge, attitudes and competencies of nurses and the factors affecting them [16–18]. Since qualitative studies are context-based and different results are obtained in different fields, qualitative studies that explore the experiences of nurses during disasters can provide valuable insights and rich details. By gaining an in-depth understanding of these experiences, it is possible to minimize the negative effects and maximize the benefits that are important for both nurses and organizations. Investigating and comprehensively understanding the outcomes can help identify strategies to minimize the adverse consequences and enhance positive outcomes that arise from nurses’ involvement in disasters.

## Methods

### Study design

We applied conventional qualitative content analysis to explain the experiences of nurses regarding about the consequences of their presence during disasters. Qualitative content analysis design allows for multifaceted descriptions that attempt to understand and convey people’s experiences. Especially in situations where there is limited knowledge [19]. This design provides a valuable way to address critical clinical issues with a primary emphasis on participation [20].

### Participants and setting

Using purposive sampling method, this study was conducted on 20 nurses (12 women, 8 men) in the emergency departments of hospitals affiliated to Kerman University of Medical Sciences in southeastern Iran, from March 2023 to November 2023. After obtaining permission, the researchers went to the nursing offices of hospitals involved in disasters. The considered nurses were selected with the introduction and guidance of nursing managers. The participants were nurses who have been involved in disaster response efforts. Nurses who, in addition to Corona, provided care during other natural and man-made disasters, were the sample of this study. We selected nurses who were willing to express their experiences and had good expressive power to increase our understanding of the phenomenon under study. The interviews were conducted in quiet places such as the supervisor’s or educational supervisor’s room with the coordination and agreement of the participants. Also, the interviews continued until data saturation was reached. It means that no more data and new concepts of the desired phenomenon were obtained. In our study, 18 interviews were conducted until the saturation point was reached. But to ensure that no new concepts were developed, two additional interviews were conducted. Therefore, there were 20 interviews in total.

### Data collection

Data was collected by face-to-face, semi-structured interviews. All interviews were conducted by the first author, who has a 20-year clinical experience and is proficient in qualitative research. During the interviews, a set of general questions were asked, such as “What were your general experiences of being in the disaster?”, “What was the impact of your presence during disasters on your mental and physical health?”(Table 1). Follow-up questions such as” Can you explain more?” or” Can you give an example?” were used. The interviewer tried to suspend her own idea about the study topic. The interviews lasted between 30 and 60 min, and MAXQDA10 was utilized for coding, categorizing, and extracting theme. Sampling was performed with maximum variation in terms of age, gender, and the number of years of work, Duration of being during disasters, type of disasters experienced by participants, experience and job position.

### Data analysis

The qualitative content analysis approach suggested by Graneheim and Lundman was used for this study [21]. The data analysis was carried out concurrently and continuously with the data collection process. After each interview, the entire text of the interview was transcribed. In the next step, the whole text was read several times to get a general understanding of the content. The meaning units were then selected and the original codes decoded. The codes were then categorized based on their similarities and differences. Finally, the hidden content of the information was extracted.

### Trustworthiness

In order to ensure the trustworthiness of the study, we applied four key criteria throughout the research process: credibility, dependability, conformability, and transferability [22]. To enhance credibility, researchers conducted member checks with participants during data collection and analysis. This involved participants reviewing interview content, codes, and themes to ensure accuracy and reflection of their experiences. Peer checking techniques were also utilized to further support credibility through long-term engagement with the data. For dependability, all raw data, codes, and subcategories were saved for auditing purposes, with detailed notes on study procedures. To ensure conformability, researchers shared reflective manuscripts on the research topic, allowing for acknowledgment of previous experiences and understandings. Reflective thinking was also employed to set aside personal biases and perspectives. Lastly, a diverse sampling approach was used to improve the transferability of findings.

### Ethical considerations

This study has been approved by the ethics committee of Kerman University of Medical Sciences, with the code (IR.KMU.REC.1401.188). Describing the purpose of the research, obtaining written informed consent forms to participate in the study and recording their voice from all the participants, commitment to share the findings if desired, maintaining the anonymity, and the participants’ being able to withdraw at any stage of the research were the ethical considerations of this study. The study adhered to the ethical principles outlined in the Declaration of Helsinki (http://www.wma.net/en/30publications/10policies/b3/).

**Table 1:** Examples of question

1- Can you share any particular experiences or stories from your involvement in disasters that have deeply affected you or your colleagues?
2- What advice would you offer to upcoming nurses or aspiring healthcare professionals who may encounter disasters in their careers? What important lessons do you think are essential for anyone entering this field?
3- Have you observed any differences in your personal and professional relationships after participating in disaster situations? How do these experiences impact teamwork, cooperation, and communication among nurses?
4- Did you encounter any unexpected negative outcomes or difficulties as a result of your involvement in disasters? If so, how have these experiences shaped your perception of disaster response and recovery?
5- Is there anything else you would like to share or any additional insights you believe the research team should take into account for their study on this subject?

## Results

Of the 20 nurses who participated in the study, 11 were male nurses, 18 were married, and 14 had a bachelor’s degree. The mean age of the participants was 43.4 ± 6.5 years, and they had an average work experience of 17.1 ± 5.99 years. In terms of professional status, the participants consisted of 16 staff nurses, 1 nurse manager, 3 supervisors, and 4 matrons (Table 2). The reason for selecting samples from different categories of nurses was to gain a broader perspective on the topic being investigated. Nurses at different management levels and in different specialties have different experiences and insights that can contribute to a more comprehensive understanding of the subject.

**Table 2:** Characteristics ‘participants (*N* = 20)

No.	Gender	Marital status	Work experience (Years)	Position	Level of education
1	M	Married	20	Nurse	Bachelor’s degree
2	F	Married	25	Nurse	Bachelor’s degree
3	F	Married	10	Nurse	Bachelor’s degree
4	F	Married	26	Head nurse	Bachelor’s degree
5	F	Single	10	Nurse	Master’s degree
6	M	Married	10	Nurse	Bachelor’s degree
7	M	Married	22	Nurse	Bachelor’s degree
8	F	Married	23	Head nurse	Bachelor’s degree
9	F	Married	25	Nursing manager	Master’s degree
10	M	Married	20	Nurse	Bachelor’s degree
11	M	Single	10	Nurse	Bachelor’s degree
12	M	Married	18	Nurse	Bachelor’s degree
13	F	Married	22	Nursing manager	Master’s degree
14	M	Married	8	Nurse	Master’s degree
15	M	Married	20	Head nurse	Bachelor’s degree
16		Married	15	Nurse	Bachelor’s degree
17	M	Married	10	Nurse	Bachelor’s degree
18	F	Married	17	Nurse	Master’s degree
19	M	Married	19	Nurse	Bachelor’s degree
20	M	Married	12	Nurse	Master’s degree

The analysis of the interviews identified two main categories: acquiring experience in difficult conditions and overlapping of job frustrations. The main category of acquiring experience in difficult conditions encompassed several subcategories, including improved quality of care, experience and dedication in fulfilling the role, reduced stress, occupational motivation and enthusiasm, increased self-confidence, and improved social status. On the other hand, the main category of overlapping of job frustrations comprised the subcategories of physical strength deterioration, psychological and emotional distress, discontinued from supports, feelings of hopelessness, increased exposure to violence and aggression, and occurrence of family problems (Table 3). The following quotes from the participants further explain these categories and subcategories.

**Table 3:** Themes and subthemes extracted from content analysis

Theme	Sub-themes
Acquiring experience in difficult conditions	Improved quality of care
	Experience and dedication in fulfilling role
	Reduced stress
	Occupational motivation and enthusiasm
	Increased self-confidence
	Improved social status
Overlapping of job frustrations	Deterioration of physical strength
	Psychological-emotional distress
	Increased exposure to violence and aggression
	Occurrence of family problems
	Discontinued from supports
	Feeling of hopelessness

### Acquiring experience in difficult conditions

Despite the adverse consequences, nurses have consistently demonstrated their unwavering reliability as responders during disasters, often putting their own safety at risk to aid patients. They firmly believe that their presence during disasters allows them to gradually acquire competencies that yield positive and constructive outcomes. This category encompasses a range of subcategories, including improved quality of care, invaluable experience and dedication in fulfilling role, reduced stress, occupational motivation and enthusiasm, increased self-confidence, and improved social status.

### Improved quality of care

According to the participants, when nurses are competent and adequately prepared for a disaster, they are able to react and respond swiftly. As a result, patients benefit from improved access to care, while effective time management is also facilitated.

*Upon* returning *from the devastating earthquake in Zarand, a city in southeastern Iran, I immediately went to the emergency room at Hospital S-B. However, this time, I was able to triage the patients much more efficiently than I had done in the past. (Participant # 6)*

*The respiratory team consisted of highly competent and skilled nurses, collaborating closely with expert doctors. In addition to providing care and treatment, they also took on the responsibility of educating other nurses. As a result, the quality of care provided by the nurses improved significantly, and we were confident that patients would be promptly connected to ventilators without any unnecessary delays. (Participant # 15)*

### Experience and dedication in fulfilling the role

The presence of nurses in disasters provided them with valuable opportunities to test and address any skill deficiencies they may have had. As a result, they played a crucial and effective role in delivering care at various levels. Consequently, they gained a reputation as highly competent nurses with a broader skill set compared to their counterparts.

*When comparing nurses who have experienced earthquakes, floods, and other disasters with those who have not, the former tend to possess a higher level of resilience and adaptability. Regardless of the setting they are placed in, these nurses bring a sense of confidence and ease. (Participant # 20)*

*Being present during disasters, such as the COVID-19 pandemic, made us acutely aware of our knowledge gaps and motivated us to seek improvement. We specifically want to* learn *how to work with a ventilator and perform intubation, skills we had not possessed previously. This realization stem from the need to effectively care for COVID-19 patients. We are confident that this newfound knowledge would undoubtedly prove valuable for our future practice. (Participant # 5)*

### Reduced stress

Nurses recognized the critical significance of stress reduction after attaining competence. Drawing from their extensive experience in disaster care, they witnessed firsthand the profound impact that stress can have on patient outcomes and the overall quality of care. Consequently, they understood that by reducing stress, they could enhance their cognitive abilities, minimize errors, and make more robust decisions.

*Confidence in* our *skills and knowledge plays a crucial role in reducing a significant amount of stress. Continuous learning and development further contribute to our ability to adapt to new challenges, which, in turn, helps reduce stress. (Participant #8)*

*Following the completion of* disaster *management training and specific training for the Corona response team, nurses who initially felt stressed about being deployed to affected areas willingly volunteered to provide care during disasters. (Participant #3)*

### Occupational motivation and enthusiasm

The experience of being present during disasters and the pursuit of necessary competencies sparked a deep interest and motivation among nurses in their work. This motivation enabled them to endure even the most challenging working conditions and effectively deal with their responsibilities.

*By saving patients’ lives and experiencing the profound sense of usefulness that comes with it, my motivation has* significantly *increased. This newfound motivation has instilled in me a strong readiness to go to any part of the country to provide assistance to patients in need. (Participant #5)*

*The nurse’s role in aiding earthquake victims was often likened to that of an angel. This recognition of* the *nurse’s profound impact and the belief that I had done a great job served as a powerful motivator to continue my work. (Participant #17)*

### Increased self-confidence

According to the participants, their work experience in disaster had a profound impact on the improvement of their skills. This improvement, in turn, led to increased self-confidence, enabling them to provide independent care, initiate patient diagnoses, intervene effectively, and make informed decisions about patient care. The nurses expressed that the more they worked in disasters, the more their confidence grew.

*Repetition of a task tends to increase confidence and competence. This is particularly true for specialized tasks like disaster triage, which differs significantly from routine practice. Having previously performed disaster triage multiple times, I was confident and certain in my ability to make quick and accurate decisions about the priority of patients. (Participant #19)*

Nurses often encounter various challenges and difficulties on the path of obtaining competencies. However, through repetition and dedicated practice, nurses can strengthen their self-confidence and enhance their knowledge and learning. This continuous repetition allows nurses to refine their skills and perform their caregiving functions effectively.

*In situations where* casualties *with cardiopulmonary arrest arrived at the Emergency Department, it was common for nurses to find themselves alone and responsible for initiating life-saving measures. With confidence in my knowledge and abilities, I took immediate action by calling the code and swiftly initiating CPR. However, many nurses tend to wait for the doctor’s arrival due to a lack of confidence in their own abilities. (Participant # 13)*

### Improved social status

The participants expressed that despite enduring numerous hardships during disasters, they never regretted being present to provide care. The efforts and dedication of the nurses were highly praised by the patients they served. They considered the attention and support of the media, society and managers as a sign of improving their social status and this led to higher job satisfaction for the nurses.

*The media coverage highlighting the discharge of COVID-19 patients and the crucial role played by nurses in saving lives has resulted in people considering nurses as heroes. The dedication and sacrifice of tens of thousands of nurses who risked their lives on the front* lines *to provide care have earned them immense respect and admiration in society. Being recognized as a hero by both the public and within one’s own profession is a great honor for me. (Participant # 14)*

Therefore, nurses have significantly improved society’s perception of the nursing profession through their selfless sacrifices, willingness to risk their lives, and utilization of their skills and knowledge during disasters.

*Managers played a crucial role by providing nurses with the necessary equipment and comfortable accommodations, which greatly supported their work. People in the community showed immense respect and followed our advice, recognizing our expertise and dedication. The outpouring of praise for the healthcare team on social media further reinforced the positive impact of their efforts. This recognition improved the social image of nurses. (Participant #17)*

### Acquiring experience in difficult conditions

Nurses often face occupational consequences that are inherent to their profession. However, during disasters, the impact on their bodies and minds becomes even more pronounced. This can be seen across several subcategories, including deterioration of physical strength, psycho-emotional distress, increased exposure to violence and aggression, occurrence of family problems, discontinued from supports, and feeling of hopelessness.

### Deterioration of physical strength

When analyzing physical strength, it becomes evident that many nurses sacrificed their own health during disasters. The demanding circumstances, including extended work hours without breaks, prolonged standing, inadequate sleep and nutrition, heavy lifting, and the overall physical demands of their role during such crises, often exceeded their physical endurance. Consequently, their health becomes compromised due to the strain placed on their bodies.

*During a long shift, I often find myself juggling multiple tasks simultaneously. I not only have to meet the needs of patients but also interact with families who are seeking updates and* reassurance. *Moreover, I may face requests from multiple doctors for various procedures, further adding to my workload. one nurse alone is insufficient to handle all the work. As the shift progresses, I may experience physical discomfort such as backpain and foot ache.* (Participant # 1)

*After enduring 12–16-hour workdays, I experienced profound fatigue, to the point where I could almost fall asleep while standing. The physical strain of such long hours resulted in* pervasive *pain throughout my body. (Participant # 9)*

During disasters, nurses might encounter hazardous substances, including chemicals and radiation, as well as face challenges such as dehydration, heat stroke, and extreme cold. These exposures can give rise to respiratory issues, skin irritation, and other health problems.

### Psycho-emotional distress

The participants in the study reported experiencing both short-term and long-term consequences of mental-psychological injuries. Some participants expressed feelings of helplessness, isolation, and rumination as immediate reactions. In the initial days following the disaster, many experienced a sense of shock. However, even years later, the aftermath left lasting effects, including traces of depression and mental-psychological disorders among individuals.

*Currently, many of us are experiencing challenges with our mental health. It seems that we all have been affected by various mental stressors, and our tolerance levels have* significantly *decreased. This has led to difficulties in tolerating others, including our own family members. It is important to note that this situation is not unique to me; my colleagues are also facing similar struggles. (Participant # 14)*

Furthermore, the participants experienced emotions such as sadness, depression, hopelessness, anxiety and shock.

*As I entered the hospital, an overwhelming sense* of *sadness enveloped my heart. I felt an intense urge to cry. Memories of patients who had been alive just the day before, but were no longer with us, flooded my mind. The uncertainty surrounding the patients’ conditions added to my distress. The thought of something happening to them weighed heavily on my mind. Each shift was filled with constant worry and anxiety, not only for the patients but also for myself. (Participant #4)*

### Increased exposure to violence and aggression

During times of disasters, when individuals are gripped by fear, shock, and uncertainty, there is a disturbing trend of increased violence against nurses. The participants in the study perceived this aggression and violence as a significant threat to their safety and overall well-being. Such acts of violence have led to a range of negative psychological consequences for nurses, including feelings of humiliation, guilt, anxiety, depression, fear, hopelessness, burnout, and helplessness.

*Despite our best efforts*, there *was a perception among others that we did not perform well. This led to hurtful comments, which only added to our exhaustion. (Participant # 1)*

*In those challenging conditions, we endured numerous hardships, including inadequate food, sleep* deprivation, *long shifts, and low salaries. However, what remains etched in my mind is the way companions, who were grieving the loss of their loved ones, expressed their frustration through obscenities and acts of violence towards us. These behaviors had a lasting impact on me. (Participant # 13)*

### Occurrence of family problems

One of the adverse effects of nurses’ presence during disasters was the strain it placed on their family relationships. Being away from their families and unable to fulfill their family responsibilities caused discomfort and led to protest from family members.

*During a critical period when my family needed me the most, I made the difficult decision to be away from them. Reflecting on this, I question whether I have been able to fulfill my* role *as a good father to them. (Participant # 11)*

In contrast, the frequent absence of nurses from the family environment was perceived as a form of evading life responsibilities, placing an increased burden on their spouses and other family members. This dynamic had negative effects on family relationships, leading to the emergence of conflicts among them.

*My daughter, who suffers from epilepsy, experienced a severe epileptic attack while I was away during the Zarand earthquake. My wife holds me responsible for this incident, as my daughter heavily relies on me and I had been absent for an extended period. The weight of the situation was unbearable for her. (Participant # 3)*

### Discontinued from supports

Participants recognized that the COVID-19 pandemic brought about distinct challenges for nurses beyond the hospital setting. They highlighted various social effects, such as the experience of stigma, tense interactions with others, and feelings of isolation and loneliness.

*After leaving the hospital, I noticed* that *my cousin started to distance herself from me. It was difficult to experience this rejection, as I felt others were avoiding me due to my role as a nurse constantly caring for COVID-19 patients. (Participant # 17)*

*I had a sense of being caught in the midst of plague, feeling as if I had contracted the disease myself. When I* hailed *a taxi to go to the hospital, I had to provide an address close to the hospital just to convince the driver to take me. This experience was truly distressing and painful. (Participant # 12)*

### Feeling of hopelessness

Nurses felt weak and unable to provide care due to reasons such as excessive demands, lack of resources, and the need to provide care in challenging conditions. This feeling of helplessness made them feel hopeless.

*As family members cried and embraced their loved ones, I stood there, filled with a mix of astonishment and* sorrow. *In that moment, I questioned the purpose of my own existence, knowing that my role was solely to extract lifeless bodies from beneath the rubble. (Participant # 16)*

*It was undeniably disheartening, as we encountered numerous cases of patients for whom we were* unable *to provide any assistance. The lack of sufficient equipment and medicine only exacerbated the frustration we felt in our efforts to help them. (Participant # 3)*

## Discussion

The data analysis revealed two main categories: overlapping of job frustrations and acquiring experience in difficult conditions. The research findings indicated that nurses experienced both positive and negative consequences by being present during disasters and striving to acquire the necessary competencies to respond effectively. The positive consequences propelled them towards professional growth and kept them motivated, while the negative consequences hindered their ability to fulfill their roles effectively.

One of the positive consequences was the improvement of the quality of care. Said et al. emphasized the crucial role of competent nurses in ensuring patient safety, reducing errors, and enhancing the overall quality of care [23]. Another study demonstrated that competent nurses, through their timeliness and quick response, instilled a sense of satisfaction and hope among the injured [24]. Additionally, in a study titled “key aspects of access to healthcare during disasters,” researchers identified competent nurses as a vital strategy for enabling faster access to healthcare, effective organization and planning, and the active participation of all segments of society in disaster response [25].

The participants in the study expressed that their professional self-confidence significantly increased as a result of their involvement in disasters. This finding aligns with the results of a study titled “protecting the psychological well-being of staff exposed to disasters: a qualitative study.” In this study, one of the categories was the positive consequences of nurses’ experiences during disasters. These included enhanced morale and self-confidence, a greater appreciation for life, increased emotional maturity, increased compassion, empathy, and understanding of individuals facing challenging circumstances [26]. Studies have shown that high levels of professional self-confidence among nurses enhance safe nursing practices, positively influence patient care, and contribute to successful career outcomes [27, 28]. Nurses with higher self-confidence exhibit more positive behaviors towards patients and family members, are more proactive in emergency situations, and show increased competence in decision-making and clinical skills. Additionally, self-confidence is associated with better interaction with patients, improved self-esteem, resilience, and reduced levels of anxiety and stress, especially during challenging times like disaster [28].

In addition, Eubank et al. highlighted self-confidence as one of the outcomes of professional development. Continuous professional development plays a vital role in improving competence, expanding knowledge, and fostering professionalism in nursing care delivery. It serves as a pathway for enhancing nurses’ competence [29].

Hayter et al. conducted a study on nurses’ experiences during natural disasters, which also reported an increase in self-confidence among nurses after their involvement in such events, corresponding with the findings of our study. Nurses in that study demonstrated improved competence in their caregiving role, leading to a greater sense of satisfaction compared to their counterparts [1].

A unique finding in this study was the enthusiasm and motivation of nurses to provide assistance despite the challenges they faced during disasters. Uzunbacak et al. also found in their study that nurses’ commitment to people and their profession served as a driving force that propelled them to the front lines of disasters [30]. From this perspective, nurses emerge as invaluable angels who shoulder the immense burden of disasters with unwavering commitment. Beyond their roles within hospitals, they faced the devastating consequences and colossal loss of life. Their efforts to remain strong for patients, themselves, and others in terrifying environments revealed their resilience, sense of duty, and willingness to make sacrifices. Consequently, it becomes evident that the positive consequences of nurses’ presence during disasters and their pursuit of competencies are interconnected like links in a chain, with each aspect contributing to the improvement of the others.

During disasters, the social standing of nurses elevates due to their crucial role in response and care. Their continuous presence and application of specialized knowledge and skills to mitigate health risks establish them as dependable responders [31]. As a result, their dedication, compassion, and unwavering commitment to patient care, even in the face of challenging circumstances, can enhance the public perception of them.

The participants in this study shared their firsthand experiences of suffering from physical injuries, either themselves or their colleagues, as a result of being present during disasters. In line with the findings of the current study, a qualitative study revealed that nurses often sustained physical injuries during disasters due to inadequate rest, improper nutrition, and insufficient fluid intake [32]. Additionally, Kim et al. highlighted that provision of care for extended hours, the physical burden and fatigue resulted from excessive workloads [33]. Furthermore, another study explored the effects of stress on nurses’ physical health during disasters. Stress frequently manifested in digestive problems, muscle pain, fatigue, headaches, sleep disturbances, and even led to the use of sleeping pills, antidepressants, and pain killers [34].

Psychological injuries experienced by nurses during disasters can significantly impact their ability to work, leaving them unmotivated and unhappy. Various studies have demonstrated that nurses often encounter psycho-emotional disorders and a wide range of problems during such events [1, 35]. These challenges can severely disrupt the delivery of high-quality care.

One of the common consequences experienced by nurses during disasters is a sense of hopelessness and the feeling of being unable to effectively treat and assist those in need. Providing care during disasters becomes exceptionally difficult [36], which has resulted in nurses feeling dissatisfied with their roles, aligning with the findings of our study. In a study by Scrymgeour, participants highlighted the lack of organizational support during disasters as a major source of frustration. They described feeling abandoned by managers and experiencing a lack of presence and availability during disasters, which they perceived as a lack of organizational support. These conditions contributed to feelings of despair and hopelessness [37].

However, it is important to note that nurses in Iran prioritize motivational factors such as trust in and seeking help from God when attending to disasters due to the religious beliefs prevalent in the country. Therefore, despite the lack of support, their greatest source of disappointment was feeling inadequate in providing the highest quality assistance. This study also revealed that nurses experienced feelings of discontinue from supports during the COVID-19 pandemic and encountered difficulties when interacting with relatives, friends, and society. They exhibited symptoms of depression, anxiety, loneliness, self-doubt, and a lack of self-worth. According to the findings of Kackin et al., fear, uncertainty, and stigma are common in biological disasters. The nurses in their study not only worried about the deterioration of their physical and mental health but also feared transmitting the virus to their families, friends, and others around them, leading them to prefer isolation [38]. As a result, nurses, who were hailed as heroes, faced stigmatization by certain segments of society due to the perceived potential for transmitting the virus.

In the present study, nurses sometimes experienced disruptions in their family relationships due to the demands of their work. The challenges nurses face in balancing their family responsibilities and work obligations during disasters can lead to conflicts and difficulties [1, 39]. It is crucial for managers to create a supportive environment for nurses’ families, as this can contribute to improved mental health and reduced concerns among nurses.

Studies have also indicated an increase in violence against nurses during critical situations and disasters, which is consistent with the findings of the present study [40, 41]. Limited interaction with family and work schedules that interfere with family responsibilities can heighten the risk of family tensions for nurses [42]. When nurses are unable to participate in family events or spend quality time with their loved ones, it can lead to decreased job performance and increased stress [43]. To address these conflicts, healthcare organizations and policymakers should implement strategies such as providing flexible work schedules, offering counseling services, and providing financial and welfare support to nurses’ families during disasters. Additionally, nurses should be encouraged to develop coping mechanisms and self-care strategies that help them balance their work and family responsibilities.

Nurses reported experiencing various forms of violence during disasters, including violence from service recipients, managers, colleagues, doctors, and society. These types of violence against nurses have been highlighted as important concerns in other studies as well [42, 44]. Of course, the presence of nurses during disasters can help them in emotional management by increasing their preparedness and strengthening their resilience. Therefore, presence during disasters can bring benefits for nurses [37].

## Conclusion

The findings of this study reveal that nurses who are present during disasters face a range of both negative and positive consequences. These consequences can impact their physical, psychological, social, professional, and familial well-being. Furthermore, these consequences can have significant effects on patients, managers, and the overall health system. Understanding the implications of nurses’ presence during disasters is crucial for healthcare managers. It enables them to recognize and implement the necessary programs and interventions to prevent negative consequences and promote positive and desirable outcomes for all stakeholders involved.

## Limitations

The findings of this study provide valuable insights into the experiences and perspectives of nurses in southeastern Iran. However, to generalize these findings and apply them to nursing practice more broadly, it is important to validate them through studies involving other nursing populations. Conducting further research in different regions and contexts will enhance the robustness and generalizability of the results.

Nevertheless, this study offers a rich and comprehensive description of the consequences of nurses’ presence during disasters and their commitment to acquiring competencies. It contributes to the existing knowledge of nursing in Iran and can also serve as a valuable resource for understanding the experiences of nurses in other regions.

## Data Availability

The datasets used and/or analyzed during the current study are available from the corresponding author on reasonable request.

## Declarations

At the request of the ethics committee, the present study was conducted under the Declaration of Helsinki and the Committee on Publication Ethics (COPE). To observe ethical considerations, the participants were provided with comprehensive information about the study goals and process. All participants provided written informed consent in all stages of the study. The participants were voluntarily enrolled, and they could withdraw from the study at any time. No names were written down or mentioned during the interviews. The interview recordings were deleted after transcription. The interviewees’ and the respondents’ confidentiality was maintained throughout the research process from data collection to publication.
